# Detecting traces of consciousness in the process of intending to act

**DOI:** 10.1007/s00221-016-4600-1

**Published:** 2016-02-26

**Authors:** Ceci Verbaarschot, Pim Haselager, Jason Farquhar

**Affiliations:** Center for Cognition, Donders Institute for Brain, Cognition and Behaviour, Radboud University Nijmegen, PO Box 9104, 6500 HE Nijmegen, The Netherlands

**Keywords:** Action, EEG, ERD, Intention, LRP, RP

## Abstract

An intention to act has different onsets when it is measured in different ways. When participants provide a self-initiated report on the onset of their awareness of intending to act, the report occurs around 150 ms prior to action. However, when the same participants are repeatedly asked about their awareness of intending at different points in time, the onset of intending is found up to 2 s prior to action. This ‘probed’ awareness has its onset around the same time as the brain starts preparing the act, as measured using EEG. First of all, this undermines straightforward interpretations about the temporal relation between unconscious brain states and conscious intentions and actions. Secondly, we suggest that these results present a problem for the view that intentions are mental states occurring at a single point in time. Instead, we suggest the results to support the interpretation of an intention to act as a multistage process developing over time. This *process of intending* seems to develop during the process of acting, leaving reportable traces in consciousness at certain points along the road.

**Electronic supplementary material:**

The online version of this article (doi:10.1007/s00221-016-4600-1) contains supplementary material, which is available to authorized users.

## Introduction

Many researchers have aimed to clarify the role of our conscious intention to act in voluntary action initiation. Libet et al. (1983) were among the first to use neuroscientific methods to investigate the nature and temporal order of the processes of mind that produce the experience of will and those that produce the action. They found that the neural preparatory processes for action (i.e. readiness potential) preceded the act by about 550 ms, whereas the onset of the intention to act was reported around 200 ms prior to action. In other words, the neural preparation for action preceded the conscious intention to act by about 350 ms. According to Libet et al., this suggests that the brain starts preparing an act before it is intended consciously by a person. If this is true, it could (and has been taken to) mean that our conscious intention to act does not play a causal role in action initiation (Libet et al. 1983; Filevich et al. 2013; Soon et al. 2008).

The results of Libet et al. (1983) have been replicated several times (Haggard and Eimer 1999; Keller and Heckhausen 1990; Trevena and Miller 2002; Verbaarschot et al. 2015), and their experimental design has been widely applied (Bai et al. 2011; Bode et al. 2011; Filevich et al. 2013; Soon et al. 2008). In these Libet-type experiments, the onset of an intention to act is typically measured through a self-initiated report by the participant. Often, the participant is instructed to watch some variant of a clock and remember its spatial configuration at the time they first feel their intention to act. The remembered configuration is reported after the performance of each voluntary movement.

To the best of our knowledge, Matsuhashi and Hallet (2008) are the only ones to have used a different approach to measure the onset of an intention to act. Instead of relying on self-initiated post-action reports, they used auditory probes to initiate a real-time intention report prior to action performance. Using this method, the onset of the intention to act was measured up to 1.42 s prior to movement, much earlier than the 0.2 s of Libet et al. (1983). These results could be taken to contradict each other as a single intention to act apparently can have different onsets. Alternatively, they might represent different stages in the process of intending. However, up till now, the question of whether the reported onsets of intending indeed differ significantly between the Libet and Matsuhashi task within the same individual has not been explored.

We developed a within-subject experiment in order to investigate whether different ways of measuring (i.e. self-initiated report vs. auditory probing) lead to different onsets of intending. We expected the onset of intending measured with auditory probes (Matsuhashi task) to significantly precede the onset of intending measured with self-initiated reports (Libet task). Furthermore, we investigated during which phases of the neural preparation for action the measured onsets of intending arise. We measured three neural signatures of the preparation for voluntary movement using electroencephalography (EEG): the readiness potential (RP), lateralized readiness potential (LRP) (Kornhueber and Deecke 1965; Shibasaki and Hallett 2006), and the event-related desynchronization (ERD) in the alpha and beta band over the motor cortex (Pfurtscheller and Aranibar 1979). These signatures have been successfully used for the single-trial prediction of the onset of voluntary movement in previous research (Bai et al. 2011; Blankertz et al. 2006; Lew et al. 2012; Schneider et al. 2013).

The results of our experiment are interpreted within a conceptual framework that is an extension of the what, when, and whether model proposed by Brass and Haggard (2008). Within this framework, results of studies like that of Libet et al. (1983) and Matsuhashi and Hallett (2008) complement rather than contradict each other. In fact, they seem to suggest that before a participant is able to provide a self-initiated report of intending their act, some form of action-related awareness is already present and can be revealed by using external probes. We suggest that these results and those of previous research support the interpretation of an intention to act as a multistage process developing over time (Dennett 1991; Uithol et al. 2014).

## Experimental methods

We combined adapted versions of the Libet and Matsuhashi task into a within-subject experiment. We believe that intended actions generally involve some form of reasoning about the current situation and relevant background information and usually lead to some observable effect for which the agent can take responsibility (Mecacci and Haselager, submitted). In the original Matsuhashi task, the acts did not involve any reasoning and did not have any effect. Therefore, we made some changes to the original task design. Inspired by Grey Walter (Dennett 1991, p. 167), we instructed our participants to watch slides on a computer screen. The participants could proceed to a new slide by pressing a button whenever they wanted to. With this everyday task, we created a more realistic experimental setting since the acts could be made for a reason (where the simplest one is being bored of the current slide) and have an effect (presenting a new slide on the computer screen). Furthermore, participants did not perform their actions with a certain frequency, but performed one action on each trial in order to enhance the level of spontaneity. Finally, in our versions of the Libet and Matsuhashi tasks, participants were free to choose *what* action to perform (a left or right hand button press) and *when* to perform it.

### Participants

Twelve healthy volunteers (23 ± 4 years old, 7 females, 10 right-handed) were tested. All participants had normal or corrected-to-normal vision and gave informed consent. The study was conducted in accordance with the ethical standards provided by the 1964 Declaration of Helsinki.

### Apparatus

Instructions and stimuli were displayed on a 17″ TFT screen with a resolution of 800 × 600 pixels and 60-Hz refresh rate. In-ear headphones were used for auditory stimuli. The participant held one small button box in each hand and used a computer mouse to indicate the remembered clock configurations during the Libet task. The experiments were run in BrainStream^1^ (Severens 2009). EEG was recorded with a Biosemi Active 2 system using 64 Ag/AgCl active electrodes sampled at 2048 Hz placed according to the International 10/20 system (Klem et al. 1999). Electrode offsets were kept under 25-μV. Four electrooculogram (EOG) electrodes, placed in bipolar pairs above/below the left eye and on the outer sides of both eyes, were used to record the muscular activity related to eye blinks and movements. To measure muscle activity in the arms, electromyogram (EMG) electrodes were placed in two bipolar pairs on the centre of the forearm (flexor flexor pollicis longus) and on both wrists.

### Procedures

The participant was seated at a table in an electrically shielded room with the display 70 cm directly in front of them. Five experimental tasks were tested: the Libet task, Matsuhashi task, sound–response task, reaction time task, and no-action task. Whereas the Libet and Matsuhashi task were the main focus of this study, the remaining tasks served to train the participants in performing these tasks.

The experiment was split into two sessions: a behavioural session and an EEG session. The behavioural session consisted of: 5 reaction time trials and 30 Matsuhashi trials for training purposes (15 min), 2 × 20 reaction time trials alternated with 3 × 50 Matsuhashi trials for main testing (30 min), and finally 20 sound–response trials and 10 Libet trials to prepare for the EEG session (15 min). The EEG session consisted of alternate blocks of 6 × 50 Matsuhashi trials, 4 × 25 Libet trials, and 2 × 25 no-action trials (60 min).

At the end of the experiment, the participants filled in a questionnaire (15 min). The total duration of the experiment with self-paced breaks between blocks was approximately 3 h.

#### Libet task

**Fig. 1:**
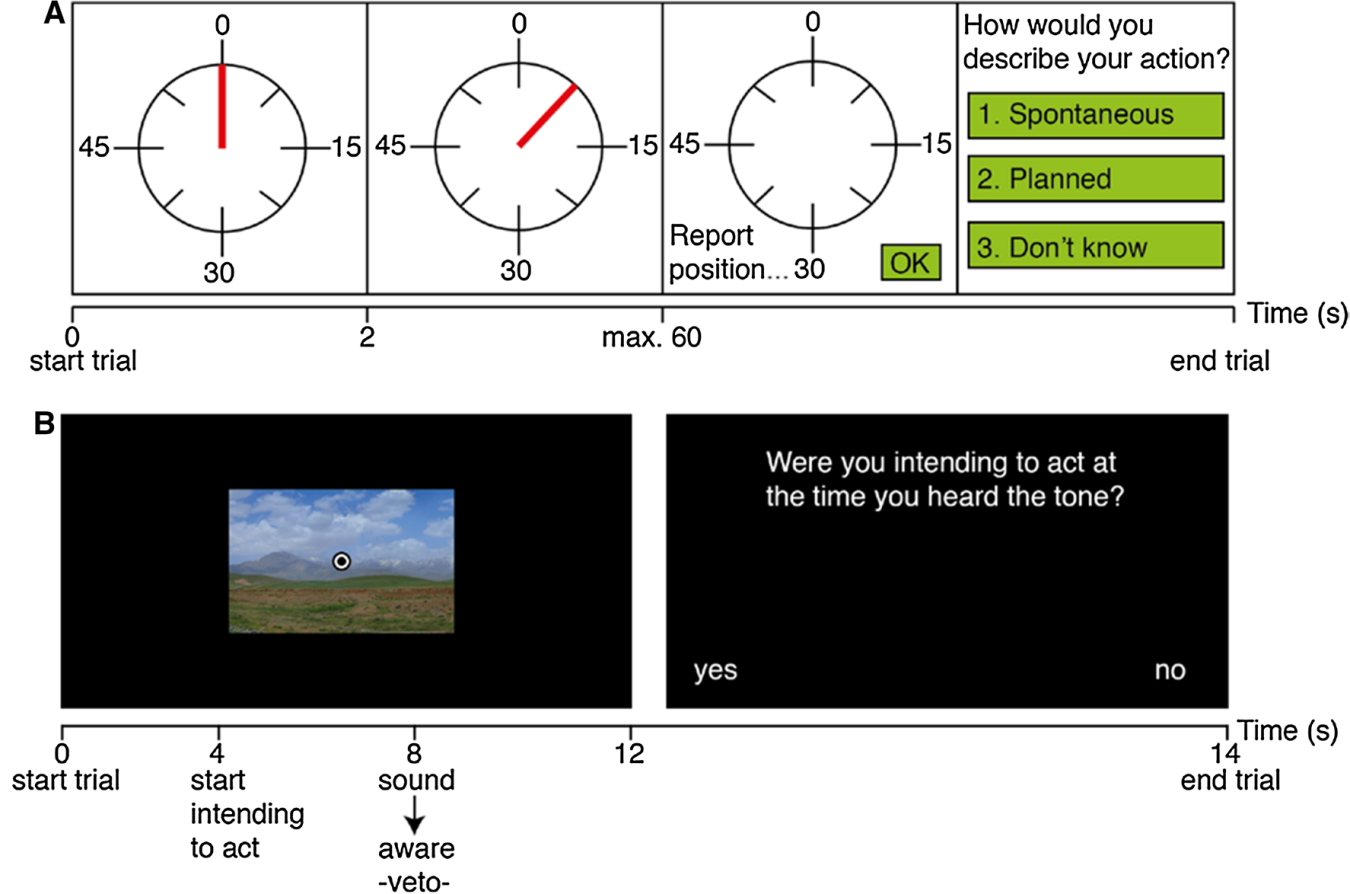
**a** Stimuli provided to a participant during a Libet trial. First, a stationary clock was presented. After 2 s, the clock started running and the participant could press a button with either their left or right hand whenever they felt the intention to do so. When a button was pressed, the participant reported the remembered clock position at the time of the intention to act. Finally, the participant reported whether the act was: ‘made spontaneously’, ‘planned’ or ‘don’t know’. **b** Sequence of events in a Matsuhashi trial in which a participant was aware of intending to act at the moment the auditory probe was presented (at 8 s) and waited for the trial to end (at 12 s). When the trial ended the participant was asked whether or not they had vetoed their act upon hearing the auditory probe

First, a stationary clock was presented for a period of 2 s. After that, it started running (see Fig. 1a). Participants were instructed to keep their eyes focused on the centre of the clock. After the clock had made one revolution, participants were free to decide *which* button to press (a left or right thumb button press) and *when* to press it. Participants were instructed to wait for the feeling of wanting to press either button to arise and remember the configuration of the clock at that time. When participants pressed a button, the clock stopped running after a random interval between 0.5 and 1 s. A new clock without a hand was presented and participants used the mouse to indicate the remembered hand position at the time they felt the intention to act. When participants were satisfied with the indicated clock position, they clicked “OK”. Finally, the question “How would you describe your action?” was displayed on the screen. Participants could answer with: (1) Spontaneous, (2) Planned or (3) Don’t know.

#### Matsuhashi task

Participants were watching slides on a computer screen^2^ (see Fig. 1b), each containing one natural landscape image (12 × 12 cm). Participants were instructed to focus on the fixation point, empty their thoughts, breathe deeply in and out and relax before intending any act. Participants could press a button with their left or right thumb to go to a new slide. While looking at these slides, a short beep was presented at a random point in time. Upon the presentation of this auditory probe, participants should: (1) veto their act if they were intending to act at the time they heard the beep and wait until the current image disappears from the screen, or (2) otherwise ignore the probe. When a participant pressed a button, the image slid off the screen in the direction of the button that was pressed. Participants could relax for 2 s between each trial. Whenever a trial ended and no button had been pressed (caused by a conscious veto or the absence of an intention to act), a question was presented, asking participants whether or not they had vetoed the act upon probe presentation.

Participants were instructed not to plan but to act as spontaneously as possible. If participants were acting too quickly (within the first 4 s of a trial) or showed a certain fixed action pattern (for example, alternate left and right hand actions), they were reminded to make sure to take their time and not plan their actions. A maximum trial length was defined in order to continue to the next trial in case of a veto. To prevent absolute predictability of the trial length, it was set semi-randomly such that 80 % of the trials lasted 11 s, 10 % lasted 12 s and 10 % lasted 13 s. In case a trial would end before participants had intended to press a button, they were instructed not to worry and continue their normal routine at the start of the new trial.

In order to optimize the probe onsets such that most occur during the interesting action intention period, an individual probe distribution was used for each participant. Initially using the training data and the updated every 50 trials, the average and standard deviation of the movement onset times were computed. A probe window was defined as 0.5 s before the average movement onset (i.e. button press) plus and minus one standard deviation. The window was shifted by −0.5 s to increase the chance that a probe was presented prior to action onset.^3^ Within this window, 70 % of the probes occur *before* and 30 % *after* the average movement onset −0.5 s.

The Matsuhashi task of the behavioural session contained many auditory probes in order to optimize the intention estimate. However, it contained very few probes in the EEG session in order to minimize auditory artefacts. Specifically, in 4 Matsuhashi blocks of the EEG session, 8 out of 10 trials probed at 10 s after trial onset whereas the remaining 2 trials were probed randomly. This increased the chance that the participant would act before probe presentation in the EEG session. The participants were not informed about these differences.

#### Training tasks

The sound–response task was similar to the Libet task; only here the participant should remember the configuration of the clock and press a button at the time they heard an auditory stimulus (the same short beep as used in the Matsuhashi task). This task was used to train a participant in remembering and reporting clock configurations for the Libet task. Furthermore, this task served as global accuracy measure of the reported clock configurations.

A simple reaction time task was used to assess the elapsed time between the presentation of an auditory probe and a button press. At the start of each trial, a fixation cross was presented for 1 s, which was followed by a black screen. At a random point in time within an interval of 3 s, a beep sound was presented. Upon hearing the sound, the participant was instructed to press a button with either the left or right thumb as soon as possible. The trials were divided into two blocks, one for left and one for right hand responses.

These trials were similar to the Matsuhashi task, with the difference that the images slided off automatically to the left or right side of the screen after a random interval of 5–5.5 s. They served to bias participants towards pressing a button roughly each 5 s without explicitly instructing them to do so.

#### Questionnaire

At the end of the experiment, the participants filled in a questionnaire on their subjective experience during the Matsuhashi task. This questionnaire aimed to find out whether the acts were made spontaneously, whether a certain action strategy was used, and whether the probes had affected decision-making.

### EEG analysis

The raw data of the Libet and Matsuhashi tasks was sliced in trials of 8 s around each button press (from 6 s before each button press until 2 s after). Each trial was labelled according to its action type: a left or a right hand action. Furthermore, the raw EEG data were down-sampled from 2048 to 128 Hz. For the Libet task, only trials in which the act was reported to be ‘spontaneous’ were used in the analysis, i.e. ‘don’t know’ or ‘planned’ trials were excluded. Furthermore, trials in which the button press occurred within 4 s of the trial start were excluded, since the baseline period might include reactions to the stimuli changes at the start of the trial. For the Matsuhashi task, only trials containing a button press were used for EEG analysis, excluding those in which the button press occurred within 4.5 s from the trial start.^4^ Trials in which a probe was presented within 4.5 s prior to the act were excluded, since they cause an additional event-related potential in the EEG, which may interfere with the baseline. To remove noise from the recorded raw EEG, the data was pre-processed in the following order:

- Linear detrending to remove slow drifts from the data
- Re-referencing to the common-average
- Rejection of bad trials (and channels) where the trial (resp. channel) power deviated more than 3.5 standard deviations from the median
- Removal of eye blinks and movements by linear decorrelation of the EEG and the EOG (Gratton 1998)

The EEG signal from 3.5 s until 2.5 s before each button press was used as a baseline for both the Libet and Matsuhashi task. Since the participants were instructed to relax and wait for their intention to act to arise during both tasks, this period was assumed to represent normal brain activity. Furthermore, we did not expect the RP or ERD signals to occur earlier than 2 s prior to action onset (Kornhueber and Deecke 1965; Shibasaki and Hallett 2006; Pfurtscheller and Aranibar 1979). As described above, only trials in which the baseline period contained resting non-artefact contaminated EEG data were analysed.

For the RP and LRP analysis, the EEG data were band-pass-filtered between 0 and 15 Hz, and any power above 30 Hz was completely removed. Next, for each electrode and trial, the average baseline signal was subtracted from the data. Finally, the data were further down-sampled to 96 Hz.^5^ For the ERD analysis, the signal amplitude in the alpha (8–12 Hz) and beta (13–30 Hz) bands was calculated using a Hilbert transform between 8 and 30 Hz. Next, the baseline (running from −3.5 until −2.5 s) was subtracted and the data down-sampled to 96 Hz. The Hilbert transform represents a signal in terms of its amplitude and phase in the target frequency range varying over time (Bruns 2004). Here we only used the amplitude at each time point allowing us to use the same methods to find the alpha/beta ERD onset as used for the RP. A spectrogram was also calculated using the short-time Fourier transform (STFT) technique (Bruns 2004), restricted to the frequency range of interest (3–40 Hz) followed by subtraction of the baseline activity, using the same baseline period as for the other analyses.

Two different measures were used to determine the time point at which the ERD and RP started for each individual participant:

1. *Eye*-*measure:* starting at the time of the button press, we looked back in time to determine the point at which five subsequent data samples were above the baseline value (similar to: Libet et al. 1983; Keller and Heckhausen 1990). For this measurement, we used only the Cz electrode as the early RP and ERD were most clearly expressed in this location (Shibasaki and Hallett 2006). Since there were no signs of lateralization within the RP or ERD at the CZ electrode, this measurement was based on the average EEG over all actions.
2. *Classifier*-*measure:* in order to get a more reliable estimate of the ERD and RP onset, we trained a regularised logistic regression classifier on baseline and action preparation data (similar to Lew et al. 2012). For this analysis, we used a slightly broader range of electrodes (Cz, C1, C2, C3, C4, FCz, FC1, FC2, FC3, FC4, CPz, CP1, CP2, CP3, CP4) in order to catch the extended brain signal correlated to motor preparation. During the baseline period (running from −3.5 to −2.5 s prior to action), we assumed that there was no neural preparation for action. For each trial, we took a 500 ms sample (running from −3 to 2.5 s prior to action) from the baseline period. These samples represented the baseline class. 500-ms Action preparation samples were taken each 10 ms between −2.5 and 0 s prior to action. The samples taken from a specific time period over all trials represented the action preparation class of that specific time period. For each time period—i.e. [−2.500, −2.000], [−2.490, −1.990], …, [−0.510, −0.010], [−0.500, 0.000]—we trained a separate classifier to distinguish between baseline samples and action preparation samples using tenfold cross-validation. The classifier input thus consisted of a vector of [15 channels, 49 time points, × trials] (see Online Resource 5 for the amount of artefact-free trials that was used for each participant). Classification performances were considered significant when they were above the binomial confidence interval (Billinger et al. 2013). The RP and ERD onset were determined by looking backward in time as the earliest time point after which three subsequent classification performances were insignificant.

The LRP was calculated for each participant using the following formula (as in Trevena and Miller 2002):

$${\text{LRP}} = \left[ {\left( {C3l - C4l} \right) + \left( {C4r - C3r} \right)} \right]/2$$

where *C*3 and *C*4 are the EEG recordings over the motor cortex of the left and right hemisphere, respectively, and *l* and *r* indicate the average EEG activity of left or right hand actions, respectively. The LRP onset was determined by looking backwards from the time point of the button press to the time point where five data samples in a row were above the baseline value (Libet et al. 1983).

## Results

### Reaction times

Whenever the measured EMG exceeded 20 µV, the muscle was assumed to be active, indicating a button was being pressed. The average difference over all participants between the button press and the onset of EMG activity was −0.002 s (SD = 0.004 s) for both the Libet and Matsuhashi task. Since this difference was so small, the button press instead of the EMG activity was used to indicate the action onset.

For the reaction time trials, the average reaction time over all participants between hearing the beep and pressing a button was 0.295 s (SD = 0.087 s). For the sound–response trials, the average was 0.365 s (SD = 0.182 s). The average difference over all participants between the actual and reported sound onsets of the sound–response trials was 0.088 s (SD = 0.037 s). An overview of all reaction times can be found in Online Resource 1.

### Onset of intending

An overview of the results on the onset of intending during the Libet and Matsuhashi task, including the onsets of the point of no return, is provided in Table 1.

**Table 1:** Overview of the intention, point of no return (PONR), alpha/beta event-related desynchronization (ERD), readiness potential (RP), and lateralized RP (LRP) onsets of both the Libet (Lib.) and Matsuhashi (Mat.) task

Subject	Intention (s)		PONR (s)	ERD (s)		RP (s)		LRP (s)	
	Lib.	Mat.	Mat.	Lib.	Mat.	Lib.	Mat.	Lib.	Mat.
1	−0.325	−1.837	−0.250	−1.820	−0.510	−1.320	−2.200	−0.270	−0.560
2	−0.048	−1.200	−0.200	−1.710	−2.030	−1.640	−0.810	−0.320	−1.370
3	−0.059	−2.478	−0.200	−1.040	−0.780	−0.760	−1.260	–	−0.400
4	−0.241	−3.249	−0.267	−2.170	−0.540	−0.950	−1.320	–	−1.350
5	−0.005	−0.758	−0.080	−0.790	–	−2.330	−0.570	–	−0.380
6	−0.063	−2.672	−0.132	−1.070	−0.510	−1.940	−0.980	−2.310	–
7	−0.135	−2.000	−0.204	−1.210	−	−2.480	−1.370	−	−2.810
8	−0.051	−2.594	−0.199	−1.340	−1.560	−0.590	−1.200	−0.310	−0.410
9	−0.074	−2.946	−0.670	−0.790	−0.990	−1.570	−2.600	−0.350	−0.910
10	−0.514	−1.662	−0.378	−2.430	−2.310	−1.750	−2.480	−0.820	−0.480
11	−0.087	−1.450	−0.199	−2.460	−1.740	−2.940	−1.230	−0.380	−0.530
12	−0.132	−3.196	−0.249	−0.510	–	−1.440	−1.680	−0.540	−0.230
Mean	−0.145	−2.170	−0.252	−1.445	−1.219	−1.643	−1.475	−0.660	−0.860
SD	0.147	0.809	0.150	0.663	0.702	0.705	0.644	0.690	0.760

The ERD and RP onsets were calculated using a classifier and the LRP onsets were calculated by eye. The mean and standard deviation (SD) are provided for each column

#### Libet task

For each Libet trial, the onset of intending to act was calculated by counting backwards from the button press. Since each button press should have been made spontaneously, the intention was assumed to arrive within 2.56 s before a button press (within one revolution of the clock). However, on some trials the angle between the 12 o’clock position and the clock hand at the time of intending was just larger than that of the clock hand at the time of the button press (for more details, see Online Resource 2). This makes sense given that the participants were on average 0.088 s late (see the “Reaction times” section) in reporting the remembered clock configurations—which translates to an inaccuracy of 122° in the hand position. To address this issue, clock hand angles up to 40° *after* the angle of the button press were treated as coming after the button press, with all others before the button press.

#### Matsuhashi task

**Fig. 2:**
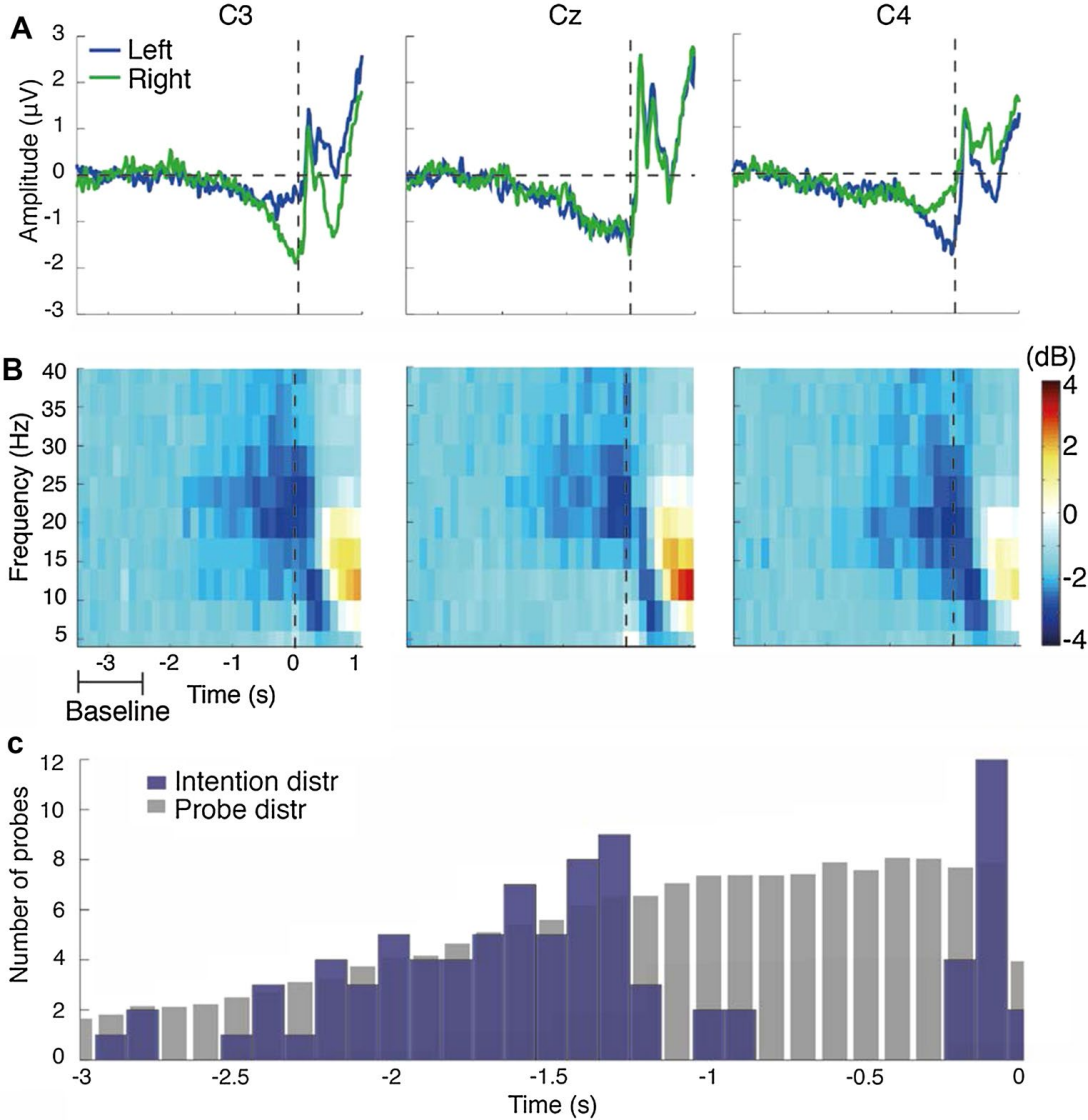
**a** Grand average of the RP for the Matsuhashi task. As can be seen in C3 and C4, the lateralized nature of the RP was clearly expressed close to action onset. **b** Grand average time–frequency representation of the data of the Matsuhashi task. The ERD in the alpha/beta range was clearly visible and seemed to start around 1.5 s prior to action onset (time zero). **c** The intention (*blue*) and probe (*grey*) distributions of participant 2. Each *bar* in the intention distribution represents the average number of times that a probe was presented and ignored at a certain point in time prior to an act. Each *bar* in the probe distribution represents the average theoretical number of probes that have been presented at a certain point in time. For example: 1 s prior to action, two probes were presented, ignored and followed by an action 1.4 s later. However, at that time a total of seven probes have been presented. This means that five probes were not followed by an act, since the participant started intending their act and performed a veto upon probe presentation (colour figure online)

In order to calculate the onset of intending to act during the Matsuhashi trials, the intention and probe distributions were calculated for each participant (see Fig. 2c for the distributions of participant 2). The intention distribution refers to the distribution of the amount of probes that were followed by a button press at a later point in time. The probe distribution refers to the amount of probes that were scheduled for presentation during the experiment. The intention distribution started to differentiate from the probe distribution close to action onset. All probes that have been followed by a veto will not appear in the intention distribution, thus causing a gap: a period of time close to action onset in which the presented probes were followed by a veto. During this gap, lots of probes were presented, but no longer ignored since the participant started intending their act. Very close to action onset, the intention distribution becomes similar to the probe distribution again. At this point of no return, although a probe was presented to a participant who was intending to act, they could no longer veto their act since the probe occurred too close to action onset. The onset of intending was estimated by fitting a sigmoid to the intention distribution (see Online Resource 3).

The questionnaire showed that 11 of the participants reported feeling relaxed or at ease during the Matsuhashi task. None of the participants used a strategy to perform their acts and 11 participants confirmed that they had made their actions spontaneously or intentionally on each trial. All subjects confirmed that they felt that they were free to choose what action they would like to perform and when they would like to perform it. Six participants found it hard to judge whether or not they were already intending their act at the moment that they were presented with a probe. Moreover, three participants reported that sometimes probe presentation induced a feeling of intending to act (see Online Resource 4 for more details).

### EEG data

For the Libet task, 82 % of the actions over all participants were reported as ‘spontaneous’ and were used for subsequent analysis. In total 10 % of the actions were reported as ‘don’t know’and 8 % as ‘planned’, all of these trials were excluded from the analysis. On average 82 (40 left and 42 right hand actions) and 156 (72 left and 84 right hand actions) artefact-free trials were included in the per-participant EEG analysis of the Libet and Matsuhashi task, respectively. The exact numbers of artefact-free trials of each participant are provided in Online Resource 5.

The RP onsets are provided in Table 1. The mean significant performance of the classifier was between 54.94 % (SD = 1.48 %) and 72.30 % (SD = 4.80 %) for the Libet task and between 53.72 % (SD = 1.09 %) and 68.59 % (SD = 5.42 %) for the Matsuhashi task. In addition to the RP onsets that were calculated for each participant, the RP onset of the grand average over all participants was also calculated using the RP eye method (see Online Resource 6). The RP onset of the grand average was found to lie at −1.938 s for the Matsuhashi task and at −2.914 s for the Libet task. The grand average over Cz, C3, and C4 for the Matsuhashi task can be viewed in Fig. 2a.

An overview of the LRP results is shown in Table 1. Participant 7 for the Libet task was excluded from this analysis as only a single left hand action was performed. Furthermore, participant 6 of the Matsuhashi task and participants 3, 4, and 5 of the Libet task were excluded as the LRPs were obscured by noise. The grand average of the LRP onset over all participants was also calculated in a similar way (for an image, see Online Resource 7). The grand average of the LRP onset was found to lie at −0.570 s for the Matsuhashi task and at −0.320 s for the Libet task.

An overview of the ERD results is shown in Table 1. The mean significant performance of the classifier used to calculate the alpha/beta ERD onset was between 55.32 % (SD = 1.52 %) and 70.91 % (SD = 6.22 %) for the Libet task and between 53.46 % (SD = 1.32 %) and 66.07 % (SD = 4.32 %) for the Matsuhashi task. The grand average plot of the Hilbert transform of the Matsuhashi task can be found in Online Resource 8. Participants 5, 7, and 12 from the Matsuhashi task were excluded from this analysis, as the ERD was not visible. The grand average spectrogram is shown in Fig. 2b.

### Comparison Libet versus Matsuhashi

Multiple paired-sample and one-sided paired-sample *t* tests were used to establish the temporal order of events prior to action performance.^6^ The alpha threshold was set to 0.001 using the Bonferroni correction (Rice 1989). The detailed results of these *t* tests can be found in Online Resource 9. These tests were performed on complete data only. For instance, in order to investigate whether the LRP arises consistently earlier than the RP, only the results of participants for which both the RP and LRP onsets could be accurately calculated were used in the analysis.

**Fig. 3:**
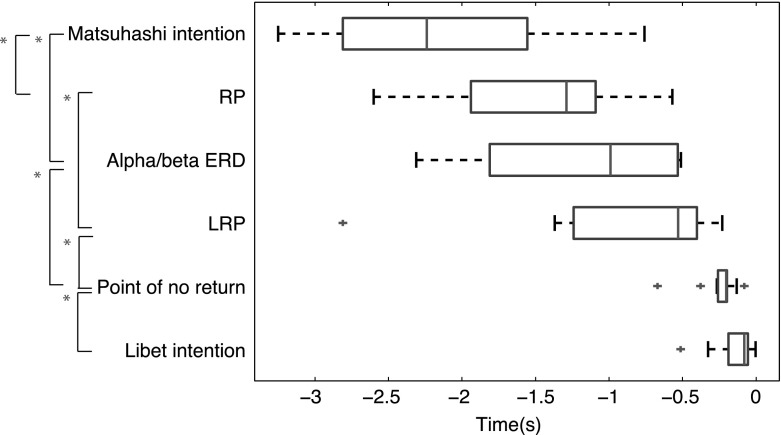
Box-and-whisker plot of the estimated RP, LRP, alpha/beta ERD and intention onsets over all participants. The *boxes* denote the first and third quartiles of the data and the whiskers extend to the most extreme data points (outliers are denoted by a *red cross*). The median is denoted by a *vertical line* inside the *box*. The RP and alpha/beta ERD *boxes* incorporate the onsets estimated by classifier for the Matsuhashi task. Similarly, the LRP *box* incorporates the estimated LRP onsets by eye for the Matsuhashi task. A *red star* indicates that the estimated onsets differed significantly between the indicated groups with *p* < 0.05 (colour figure online)

In summary, the onset of intending as measured using the Matsuhashi task seemed to arise around −2.170 s prior to action. Around the same point in time, the RP and ERD had their onset around −1.446 s prior to action. After that, the LRP had its onset around −0.760 s prior to action onset. The LRP was followed by the point of no return around −0.252 s prior to action. The onset of intending as measured using the Libet task was reported around −0.145 s prior to action. Figure 3 provides an overview of these results for the Matsuhashi task. Please note that this figure looks almost identical for the Libet task (see Online Resource 10).

Furthermore, using a within-subject cluster-based permutation test,^7^ we tested whether the RP and alpha/beta ERD data were exchangeable between the Libet and Matsuhashi tasks (using electrodes: Cz, C1, C2, C3, C4, FCz, FC1, FC2, FC3, FC4, CPz, CP1, CP2, CP3 and CP4). No significant difference was found between the RP data of the Libet and Matsuhashi task. However, one positive cluster was identified between the Hilbert transform data (8–30 Hz) of the Libet and Matsuhashi task, indicating that the alpha/beta ERD of these tasks differed significantly over time (for details, see Online Resource 8).

## Discussion

We have conducted a within-subject comparison between the Libet and Matsuhashi task in order to investigate whether different ways of measuring (i.e. self-initiated report vs. external probing) lead to different onsets of intending. Furthermore, we investigated during which phases of the neural preparation for action the measured onsets of intending occur. Our main hypothesis was confirmed: the onset of intending measured using external probes (Matsuhashi task) preceded the onset of intending measured using self-initiated reports (Libet task). This difference was 2 s on average and highly significant. Moreover, the onset of intending measured with the Matsuhashi task occurred around the same time as the onset of the RP and ERD. The onset of intending measured with the Matsuhashi task did significantly precede the onset of the LRP by 1.41 s and the point of no return by 1.92 s on average. Both the RP and ERD significantly preceded the onset of the point of no return and the onset of intending measured with the Libet task. Lastly, the RP significantly preceded the LRP by 0.80 s on average.

Before proceeding to any conclusions or interpretations of the data, there are some limitations of this study that need to be addressed. First of all, the sample size of 12 participants for this study is quite low, even though it is comparable to previous research (Libet et al. 1983; Haggard and Eimer 1999; Matsuhashi and Hallett 2008; Bai et al. 2011; Miller et al. 2011). The number of participants included in the analysis was even lower since the ERD and/or LRP of some participants was not visible or obscured by noise (as described in the “EEG data” section). However, since we performed paired-sample statistics, we conducted our analysis on the individual data of each participant. Moreover, the significance of our main results indicates that we tested a sufficient amount of participants in order to draw a valid conclusion: the onset of intending measured using external probes (Matsuhashi task) preceded the onset of intending measured using self-initiated reports (Libet task). Although our main conclusion seems valid, more data should be collected in order to clarify the more subtle effects that were found in the data. For instance, from the present data we cannot conclude whether the ERD precedes the RP or whether the point of no return precedes the intention onset as measured by the Libet task.

Secondly, the Matsuhashi task needs more validation, as it remains unclear whether the auditory probes are effective in measuring the onset of the intention to act. As described in the “Matsuhashi task” section, half of the participants found it difficult to judge whether they had intended to act upon probe presentation and three participants reported that sometimes it felt like the probes induced an intention to act. Therefore, the early onset of intending as measured during the Matsuhashi task might be due to a task-related artefact. The present results cannot exclude whether the probes might have triggered an intention to act or whether the probes induced a false-positive intention report. A false-positive intention report means that upon probe presentation, the participant believed they were intending to act, even though they in fact were not. However, since the reported onsets of intending from the Matsuhashi task occur consistently and significantly earlier in time compared to those of the Libet task, it seems unlikely that this difference in intending can be explained as a side effect of the probes alone. Moreover, the intention distributions of the Matsuhashi task show that the probes at least do not always induce an intention to act: there is a specific early time range prior to the act where the probes are ignored since the participant was not yet intending to act (for example, up to approximately 1.3 s before action as shown in Fig. 2a for Participant 2). More interestingly, there is a specific time range in which the probes caused a veto of the act. Regardless of whether these vetos indicate a false positive, triggered or true intention onset, they at least indicate that there is a specific period in time prior to action onset in which one is aware of intending to act or in which one is susceptible to external disturbances. Future research is necessary to determine what exactly is causing these vetos.

In this study, we were not aiming to validate the experimental designs of Libet or Matsuhashi, we simply wanted to compare their results in a within-subject design. For the remainder of this paper, we would like to focus on the possibility that the Libet and Matsuhashi tasks show a difference in intention onset. Under this assumption, our results show that the intention onset as measured using the Matsuhashi task significantly precedes the intention onset as measured using the Libet task. This could mean that (1) the Libet and Matsuhashi task evoke two different types of intentions which have different onsets or (2) a single intention can have two different onsets depending on its measurement procedure. Since the Libet and Matsuhashi task require the exact same motor action, it seems implausible that the corresponding intention to act would be entirely distinct. Therefore, we would like to argue that the Libet and Matsuhashi task measure the same intention, but during different phases inside a *process* of intending. The Libet task measures the point in time at which one is first able to (verbally) report *on one’s own* to be intending an act. The Matsuhashi task measures the point in time at which one is first able to report to be intending an act *whenbeing asked*. In other words, our results suggest that before a person is able to provide a self-initiated report of intending their act, some form of action-related awareness is already present and can be revealed using external probes. We suggest that these results and those of previous research support the interpretation of an intention to act as a multistage process developing over time.

### The process of intending

**Fig. 4:**
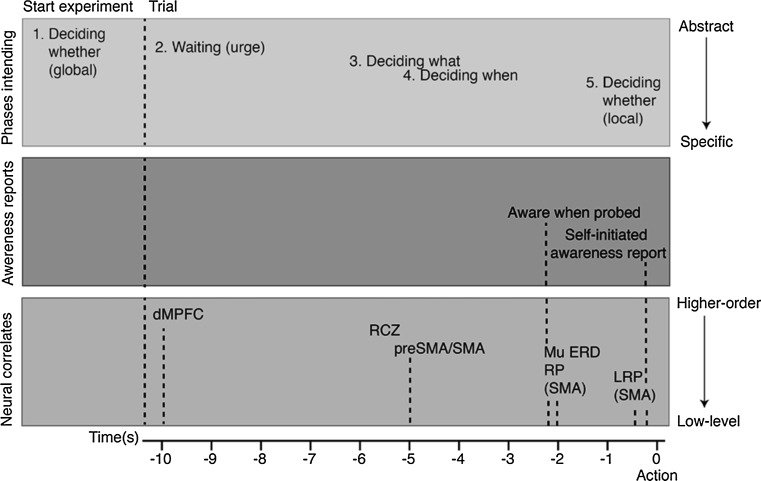
Process of intending consists of five phases (*top box*): *1* global whether decision, *2* waiting, *3* deciding what, *4* deciding when and *5* local whether decision. The phases in the process of intending run from abstract (global agreement to participate) to specific (knowing which action to perform and when to perform it). The *middle box* shows the reported onsets of intending. Phase in the process of intending can be suggested to be linked to distinct neural correlates in the brain (*bottom box*). The neural preparatory processes for action run from activity in higher cognitive areas to lower cognitive areas. *dMPFC* dorsomedial prefrontal cortex, *RCZ* rostral cingulate zone, *SMA* supplementary motor area, *RP* readiness potential, *ERD* event-related desynchronization

Major advances have been made in measuring the neural correlates of intentional action (Bai et al. 2011; Blankertz et al. 2006; Lew et al. 2012; Schurger et al. 2012; Soon et al. 2008). An influential distinction between what, when, and whether aspects of intentional action is proposed by Brass and Haggard (2008). They show that the neural preparation for an intended act seems to be distributed in both time and space as it seems to start in the frontal regions of the brain and gradually travels backwards towards the motor cortex (further described by Brass et al. 2013). In their model, Brass and Haggard describe the different phases of the neural preparation for action that seems to be correlated with the what, when, and whether decisions involved in a voluntary act. In this section, we would like to extend this model by adding information on the subjective experience of intending. Moreover, we distinguish ourselves from the original model as we interpret the what, when, and whether decisions not as different *states* of intending, but as different *phases* of intending (in line with: Dennett 2003, p. 238; Uithol et al. 2014). In other words, we find that, similar to the neural preparatory activity for action, the intention to act is also distributed in time. Below, we suggest a conceptual framework describing this *process of intending*^8^ (see Fig. 4). The framework distinguishes three aspects of intentional action: the different phases in the process of intending, the potential neural correlates of each of these phases, and the timing of the reported onsets of being aware of intending to act.

First, we will describe the five phases of intending:

1. *Global whether decision:* a global phase that starts when a participant decides to participate in the experiment.
2. *Waiting:* in between the start of a trial and the moment of deciding what action to perform and when to perform it, there is a period of doing nothing/waiting during which the urge to act comes and goes. These urges might be represented by the random fluctuations in neural activity as found by Schurger et al. (2012).
3. *Deciding what:* a phase during which specifics about the particular type of action (e.g. pressing a left vs right button) are processed
4. *Deciding when:* the moment of action is being decided when an urge to act crosses a certain threshold.
5. *Local whether decision:* a phase in which vetoing an intended act is still possible.

Second, we describe the different phases of intending in relation to the reports concerning the participants’ awareness of their intention to act. Investigations of the Matsuhashi and Libet tasks indicate that different ways of reporting provide different ‘windows of introspection’ on the intentional process. In our experiment two such windows were studied. First of all, the ‘*aware when probed*’: the participant might be able to report intending their act when they are probed by an external stimulus. Secondly, the ‘*self*-*initiated awareness report*’: at this point, the decision to act enters the participant’s awareness, which enables the subsequent report of consciously willing to act.

Third, we describe the potential neural correlates of the intentional process. It is currently not clear whether separate neural correlates of each of the five postulated phases can be identified. Yet, one can suggest links between the sequence of phases in the process of intending and various results on action preparation (Bai et al. 2011; Bode et al. 2011; Blankertz et al. 2006; Libet et al. 1983; Pfurtscheller and Aranibar 1979; Soon et al. 2008; Schurger et al. 2012; Trevena and Miller 2002). Between the (global) whether and what decision, urges to act might come and go. These urges might be represented by the fluctuations described by Schurger et al. (2012). These fluctuations might continue until one of them crosses a certain threshold and leads to a decision on the what and/or when component in the process of intending. Furthermore, the early frontal lobe activity found by Soon et al. (2008) and Bode et al. (2011) is suggested to be predictive of the subsequent act and might arise during the waiting phase, leading to the what decision in the process of intending. The activity in the pre-SMA and SMA, as found by Libet et al. (1983) and Soon et al. (2008), might be more tightly linked to the initiation of the subsequent act, representing the when decision in the process of intending (Brass et al. 2013).

Clearly, many details need to be further specified and experimentally investigated. Yet, the folk psychological interpretation of intending to act as occurring at a single point in time does not map correctly onto the results presented in this paper, as an intention to act is found to be distributed in time. It seems to be much more plausible that an intention to act is in fact a multi-stage process developing over time. If so, differentially timed reports and neural correlates are exactly what one would expect.

## Conclusion

This study presents another step in the investigation of the different phases in the process of intending and has focused on those phases that are close to action onset. The average onset of intending measured using the Matsuhashi task did not differ significantly from the RP and alpha/beta ERD onset, suggesting that these processes have their onset around the same point in time. The RP and alpha/beta ERD seem to play a part in the final stages of action preparation. However, movement intent is visible much earlier in time: starting in the frontal cortex and moving up to the (pre) supplementary motor area (Bode et al. 2011; Soon et al. 2008). The current study suggests that the process of intending develops during the process of acting, leaving traces in consciousness at certain points along the road, ultimately reaching awareness and becoming reportable. More research is needed to validate the experimental designs of the Libet and Matsuhashi tasks and to differentiate the neural processes relating to acting from those relating to intending and/or becoming conscious of intending. Our hope is that the currently emerging conceptual framework for the process of intending will help to enable a more robust interpretation of research results and set the stage for new experiments.

## Electronic supplementary material

Below is the link to the electronic supplementary material.

Supplementary material 1 (PDF 80 kb)

Supplementary material 2 (PDF 102 kb)

Supplementary material 3 (PDF 424 kb)

Supplementary material 4 (PDF 68 kb)

Supplementary material 5 (PDF 87 kb)

Supplementary material 6 (PDF 93 kb)

Supplementary material 7 (PDF 95 kb)

Supplementary material 8 (PDF 1650 kb)

Supplementary material 9 (PDF 94 kb)

Supplementary material 10 (PDF 202 kb)

## Footnotes

1 See www.brainstream.nu.

2 Images were selected from Flickr’s Creative Commons (www.flickr.com/creativecommons) and were roughly symmetrical without any animate objects.

3 During training, this time window was set by default to run from 3 to 8 s. When the variance of a participant’s movement onset distribution was <1.5 s, it was automatically set to 1.5 s in order to keep a minimum window of 3 s within which probes could be presented. If the calculated time window fell within the baseline period or outside the maximum trial length, it was automatically set to a minimum of 3.5 s and a maximum of 10.5 s, respectively.

4 This period was 0.5 s longer than the one used in the Libet task because the visual stimuli changes at the start of a Matsuhashi trial (the presentation of a new image) were larger and might cause a longer brain response than those at the start of a Libet trial (the transition from a stationary to a running clock).

5 We expected the RP/LRP signal to occur below 2 Hz and the alpha/beta ERD to occur between 8 and 30 Hz. Since the maximum component frequency that we expected to find is 30 Hz, the Nyquist rate was 60 Hz. Therefore, we did not expect to find any informative information in the EEG data above 60 Hz.

6 No significant differences were found between the ERD and RP onsets calculated by eye or by classifier (see Online Resource 9). The calculation by classifier is the more conservative and reliable measurement since it aims to detect the ERD or RP onset as the point at which the EEG signal is significantly below the baseline over all individual trials. Therefore, the onsets calculated by classifier are used as the ERD and RP onset in the rest of the article. The ERD and RP onsets calculated by eye can be found in Online Resource 6.

7 See http://www.fieldtriptoolbox.org/tutorial/cluster_permutation_timelock.

8 Note that the global whether component will not play a big role on a single-trial level in this kind of experiment since a participant agrees to perform an act on each trial by obeying the provided experimental instructions. Making this agreement leads to implementing the whether component of intending by forming a global intention to act and having an overall readiness for action. However, which action to perform and when to perform it have to be decided spontaneously on single trial.
