# Supplementary material for: Detecting traces of consciousness in the process of intending to act
Source: Exp Brain Res. 2016 Feb 26;234:1945–56. doi: 10.1007/s00221-016-4600-1 (PMC4893062; doi:10.1007/s00221-016-4600-1)
Supplement: Supplementary file 1 — Supplementary material 1 (PDF 80 kb) [file 221_2016_4600_MOESM1_ESM.pdf]

Experimental Brain Research

Supplementary material

Detecting traces of consciousness in the process of intending to act

Ceci Verbaarschot [a, 1], Pim Haselager [a] & Jason Farquhar [a]

[a] Donders Institute for Brain, Cognition and Behaviour: Radboud University Nijmegen, the Netherlands.

1 Reaction times

Subject	RT Libet (s)	Recall - sound (s)	RT Matsuhashi (s)
1	0.238	0.116	0.270
2	0.267	0.097	0.344
3	0.239	0.185	0.398
4	0.423	0.072	0.447
5	0.331	0.102	0.223
6	0.292	0.058	0.419
7	0.380	0.071	0.284
8	0.834	0.098	0.278
9	0.260	0.066	0.258
10	0.599	0.055	0.205
11	0.309	0.072	0.229
12	0.206	0.059	0.183
Mean	0.365	0.088	0.295
SD	0.182	0.037	0.087

Table 1 *Average reaction times (button press – start trial) of the sound-response and reaction time trials. Recall - sound, indicates the average difference between the reported and actual sound onset during the sound-response trials*

[1] Corresponding author. Address: Center for Cognition, Donders Institute for Brain, Cognition and Behaviour, Radboud University, PO Box 9104, 6500 HE Nijmegen, the Netherlands. Phone: +31-2436-15606. E-mail address: c.verbaarschot@donders.ru.nl (C.S. Verbaarschot).
