# Supplementary material for: Detecting traces of consciousness in the process of intending to act
Source: Exp Brain Res. 2016 Feb 26;234:1945–56. doi: 10.1007/s00221-016-4600-1 (PMC4893062; doi:10.1007/s00221-016-4600-1)
Supplement: Supplementary file 2 — Supplementary material 2 (PDF 102 kb) [file 221_2016_4600_MOESM2_ESM.pdf]

Experimental Brain Research

Supplementary material
Detecting traces of consciousness in the process of intending to act

Ceci Verbaarschot [a, 1], Pim Haselager [a] & Jason Farquhar [a]

[a] Donders Institute for Brain, Cognition and Behaviour: Radboud University Nijmegen, the Netherlands.

2 Reported intention onsets of the Libet task reflect action onset

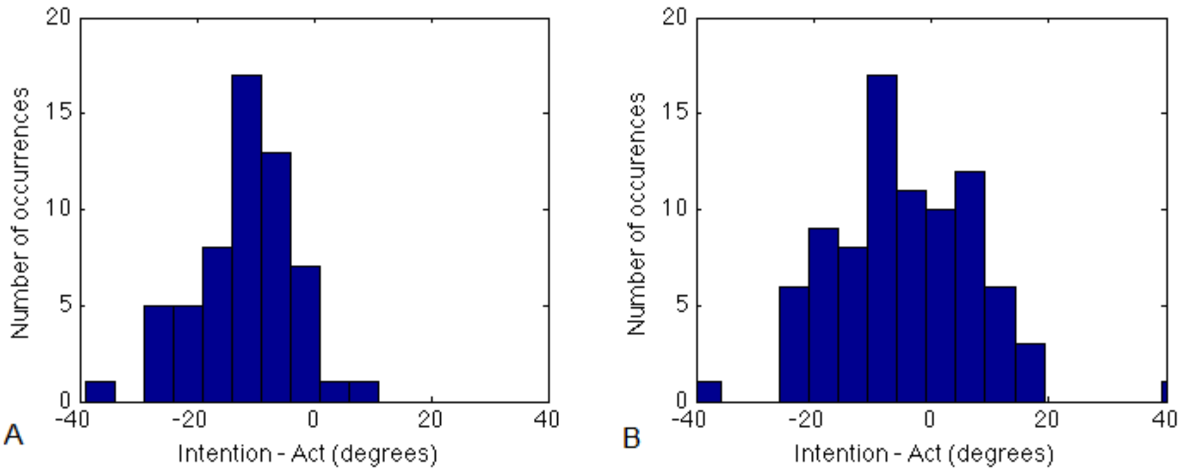

Fig. 1 *Histogram of action onset versus reported onset of intending of the Libet task for **A**. participant 11 and **B**. participant 6. The x-axis denotes the difference in degrees between action onset (0 degrees) and the reported onset of intending. A negative difference indicates that the intention was reported prior to action onset. Participant 11 reported most intention onsets to have occurred prior to action onset, whereas participant 6 reported almost half of his intentions to act to have occurred after action onset*

Intuitively speaking, most reported intention onsets should occur prior to action onset, since the thought of wanting to act is assumed to come to mind prior to action performance (as can be seen in Fig. 3A, for example). Surprisingly, around 28% of the onsets of intending over all participants were reported to occur *after* action onset. In contrast to Figure 3A, the reported intentions in these cases seem to surround the button press rather than precede it (see Fig. 3B, for example). What participants report as the onset of intending seems to be more or less equivalent to action onset, with a certain precision error. Moreover, since the point of no return was found to precede the onset of intending significantly, these intentions seem to be related more strongly to action performance than action initiation. However, the coincidence of the reported onsets of intending and the measured onsets of acting seems quite intuitive since a participant is instructed to perform spontaneous actions without any preplanning or focus on when to act. Thus, although the reported intention onsets still occur prior to action onset on average, it is important to keep in mind that these onsets do not seem to differ much on a single trial level.

[1] Corresponding author. Address: Center for Cognition, Donders Institute for Brain, Cognition and Behaviour, Radboud University, PO Box 9104, 6500 HE Nijmegen, the Netherlands. Phone: +31-2436-15606. E-mail address: c.verbaarschot@donders.ru.nl (C.S. Verbaarschot).
