# Supplementary material for: Detecting traces of consciousness in the process of intending to act
Source: Exp Brain Res. 2016 Feb 26;234:1945–56. doi: 10.1007/s00221-016-4600-1 (PMC4893062; doi:10.1007/s00221-016-4600-1)
Supplement: Supplementary file 3 — Supplementary material 3 (PDF 424 kb) [file 221_2016_4600_MOESM3_ESM.pdf]

Experimental Brain Research

Supplementary material
Detecting traces of consciousness in the process of intending to act

Ceci Verbaarschot [a, 1], Pim Haselager [a] & Jason Farquhar [a]

[a] Donders Institute for Brain, Cognition and Behaviour: Radboud University Nijmegen, the Netherlands.

3 Calculation intention onset and point of no return

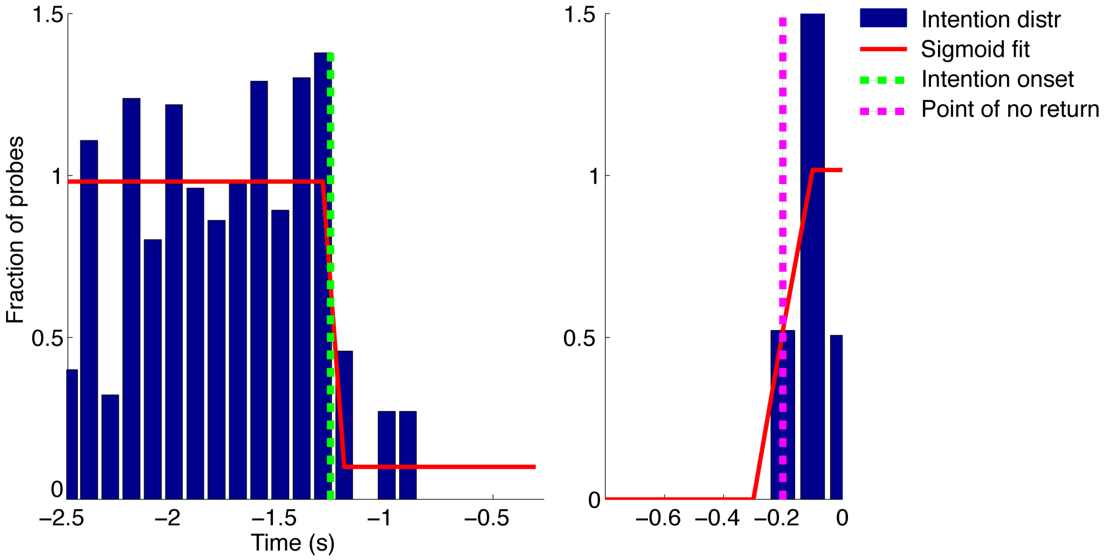

***Fig.** 4 Estimated intention onset (green) and point of no return (pink) of participant 2 using a sigmoid fit (red). The blue bars display the normalized intention distribution excluding (left plot) or including (right plot) the bins close to action onset.*

The sigmoid is of the form: $sigmoid = \frac{a(1)+a(2)}{1+e^{\frac{-x-a(3)}{a(4)}}}$

Where a(1): the earliest time point on the horizontal asymptote, a(2): the latest time point on the horizontal asymptote, a(3): the point of inflection and a(4): the width of the inflection. All these variables are estimated using the matlab nlinfit function (see mathworks.com/help/stats/nlinfit.html).

[1] Corresponding author. Address: Center for Cognition, Donders Institute for Brain, Cognition and Behaviour, Radboud University, PO Box 9104, 6500 HE Nijmegen, the Netherlands. Phone: +31-2436-15606. E-mail address: c.verbaarschot@donders.ru.nl (C.S. Verbaarschot).
