# Supplementary material for: Detecting traces of consciousness in the process of intending to act
Source: Exp Brain Res. 2016 Feb 26;234:1945–56. doi: 10.1007/s00221-016-4600-1 (PMC4893062; doi:10.1007/s00221-016-4600-1)
Supplement: Supplementary file 4 — Supplementary material 4 (PDF 68 kb) [file 221_2016_4600_MOESM4_ESM.pdf]

Experimental Brain Research

# Supplementary material
# Detecting traces of consciousness in the process of intending to act

Ceci Verbaarschot [a, 1], Pim Haselager [a] & Jason Farquhar [a]

[a] Donders Institute for Brain, Cognition and Behaviour: Radboud University Nijmegen, the Netherlands.

## 4 Probes of the Matsuhashi task induce the feeling of wanting to act

During the Matsuhashi task, 3 participants reported that sometimes, they had the feeling that the auditory probes induced the intention to act. This could be explained by the reaction time trials as participants were instructed to press a button as soon as possible after probe presentation. This learned response mapping between probes and actions could cause a vague intention to act after each probe presentation during the Matsuhashi task. This in turn might explain the earlier onsets of intending of the Matsuhashi task compared to the Libet task.
However, since the reported onsets of intending of the Matsuhashi task occur consistently and significantly earlier in time compared to those of the Libet task, it seems unlikely that this difference can be explained as a side-effect of the probes alone. Moreover, the intention distributions of the Matsuhashi task show that the probes do not always induce an intention to act. There is a specific time range prior to the act where the probes are ignored since the participant was not yet intending their act. Also, there is a specific time range where the probes caused a veto of the act since the participant was intending to act at that point in time.
Actually, these results fit rather well in the proposed framework of intending as a process, as the use of external probes seems to enable participants to report their onset of intending before they are be able to do so on their own. In other words, although the presented probes make a participant aware of their intention to act, they do not seem to cause their intention to act.

[1] Corresponding author. Address: Center for Cognition, Donders Institute for Brain, Cognition and Behaviour, Radboud University, PO Box 9104, 6500 HE Nijmegen, the Netherlands. Phone: +31-2436-15606. E-mail address: c.verbaarschot@donders.ru.nl (C.S. Verbaarschot).
