# Supplementary material for: Detecting traces of consciousness in the process of intending to act
Source: Exp Brain Res. 2016 Feb 26;234:1945–56. doi: 10.1007/s00221-016-4600-1 (PMC4893062; doi:10.1007/s00221-016-4600-1)
Supplement: Supplementary file 5 — Supplementary material 5 (PDF 87 kb) [file 221_2016_4600_MOESM5_ESM.pdf]

Experimental Brain Research

Supplementary material

Detecting traces of consciousness in the process of intending to act

Ceci Verbaarschot [a, 1], Pim Haselager [a] & Jason Farquhar [a]

[a] Donders Institute for Brain, Cognition and Behaviour: Radboud University Nijmegen, the Netherlands.

5 Overview type of action

Subject	Libet		Matsuhashi	
	#Left	#Right	#Left	#Right
1	21	75	69	78
2	40	40	69	50
3	32	43	78	82
4	73	12	34	51
5	38	57	78	54
6	45	39	100	110
7	1	76	28	138
8	47	29	92	76
9	36	39	97	108
10	62	23	70	91
11	33	28	93	99
12	54	39	61	65
Mean	40	42	72	84
GA	482	500	869	1002

Table 3 *Overview of the number of left (#Left) and right (#Right) hand actions per participant for the Libet and Matsuhashi task. These numbers indicate the artifact free trials that were used in the RP, LRP and ERD analysis.*

[1] Corresponding author. Address: Center for Cognition, Donders Institute for Brain, Cognition and Behaviour, Radboud University, PO Box 9104, 6500 HE Nijmegen, the Netherlands. Phone: +31-2436-15606. E-mail address: c.verbaarschot@donders.ru.nl (C.S. Verbaarschot).
