# Supplementary material for: Detecting traces of consciousness in the process of intending to act
Source: Exp Brain Res. 2016 Feb 26;234:1945–56. doi: 10.1007/s00221-016-4600-1 (PMC4893062; doi:10.1007/s00221-016-4600-1)
Supplement: Supplementary file 6 — Supplementary material 6 (PDF 93 kb) [file 221_2016_4600_MOESM6_ESM.pdf]

Experimental Brain Research

Supplementary material

Detecting traces of consciousness in the process of intending to act

Ceci Verbaarschot [a, 1], Pim Haselager [a] & Jason Farquhar [a]

[a] Donders Institute for Brain, Cognition and Behaviour: Radboud University Nijmegen, the Netherlands.

6 Measurements by eye

Subject	Intention (s)		PONR (s)	ERD (s)		RP (s)		LRP (s)	
	Lib.	Mat.	Mat.	Lib.	Mat.	Lib.	Mat.	Lib.	Mat.
1	-0.325	-1.837	-0.250	-1.410	-0.160	-2.600	-2.710	-0.270	-0.560
2	-0.048	-1.200	-0.200	-1.140	-0.740	-2.530	-	-0.320	-1.370
3	-0.059	-2.478	-0.200	-0.520	-0.260	-	-2.460	-	-0.400
4	-0.241	-3.249	-0.267	-1.880	-0.090	-2.660	-1.910	-	-1.350
5	-0.005	-0.758	-0.080	-1.240	-	-	-0.810	-	-0.380
6	-0.063	-2.672	-0.132	-1.120	-0.080	-2.930	-1.160	-2.310	-
7	-0.135	-2.000	-0.204	-1.230	-	-2.870	-0.930	-	-2.810
8	-0.051	-2.594	-0.199	-2.210	-1.570	-3.060	-1.000	-0.310	-0.410
9	-0.074	-2.946	-0.670	-1.450	-0.010	-2.660	-2.540	-0.350	-0.910
10	-0.514	-1.662	-0.378	-2.790	-2.080	-2.950	-1.810	-0.820	-0.480
11	-0.087	-1.450	-0.199	-2.650	-1.380	-2.230	-2.720	-0.380	-0.530
12	-0.132	-3.196	-0.249	-0.010	-	-0.030	-3.130	-0.540	-0.230
Mean	-0.145	-2.170	-0.252	-1.470	-0.710	-2.450	-1.920	-0.660	-0.860
SD	0.147	0.809	0.150	0.810	0.780	0.880	0.840	0.690	0.760
GA	-	-	-	-3.070	-1.050	-2.910	-1.940	-0.320	-0.570

Table 1 *Overview of the intention, point of no return (PONR), alpha/beta event-related desynchronization (ERD), readiness potential (RP) and lateralized RP (LRP) onsets of both the Libet (Lib.) and Matsuhashi (Mat.) task. The ERD, RP and LRP onsets were all calculated by eye. The mean and standard deviation (SD) are provided for each column. The last row provides the grand average (GA) results of the ERD, RP and LRP. Unfortunately, the RPs of the Matsuhashi task of participant 2 and the Libet task of participant 3 and 5 were obscured by noise, which made the calculation of the RP by eye impossible. The same holds for the LRP of participant 6 for the Matsuhashi task and participants 3,4 and 5 for the Libet task. Participant 7 for the Libet task was excluded from the LRP analysis as only a single left hand action was performed. Participants 5, 7, and 12 from the Matsuhashi task were excluded from the ERD analysis, as the ERD was not visible.*

[1] Corresponding author. Address: Center for Cognition, Donders Institute for Brain, Cognition and Behaviour, Radboud University, PO Box 9104, 6500 HE Nijmegen, the Netherlands. Phone: +31-2436-15606. E-mail address: c.verbaarschot@donders.ru.nl (C.S. Verbaarschot).
