# Supplementary material for: Detecting traces of consciousness in the process of intending to act
Source: Exp Brain Res. 2016 Feb 26;234:1945–56. doi: 10.1007/s00221-016-4600-1 (PMC4893062; doi:10.1007/s00221-016-4600-1)
Supplement: Supplementary file 7 — Supplementary material 7 (PDF 95 kb) [file 221_2016_4600_MOESM7_ESM.pdf]

Experimental Brain Research

Supplementary material
Detecting traces of consciousness in the process of intending to act

Ceci Verbaarschot [a, 1], Pim Haselager [a] & Jason Farquhar [a]

[a] Donders Institute for Brain, Cognition and Behaviour: Radboud University Nijmegen, the Netherlands.

7 Grand average LRP

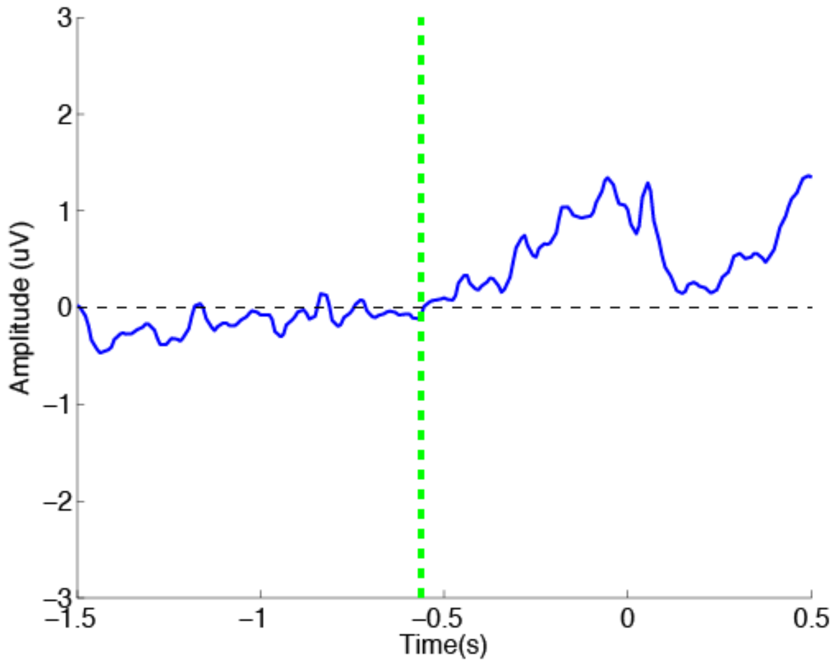

Figure 2 *Estimated LRP onset by eye (green dotted line) of the grand average over the Matsuhashi task. The positive peak prior to action performance (time 0) indicates contra-lateral activity over the motor cortex. The LRP was calculated using LRP=[(C3l-C4l)+(C4r-C3r)]/2, where C3 and C4 are the EEG recordings over the motor cortex of the left and right hemisphere respectively, and l and r indicate the average EEG activity of left or right hand actions, respectively.*

---

[1] Corresponding author. Address: Center for Cognition, Donders Institute for Brain, Cognition and Behaviour, Radboud University, PO Box 9104, 6500 HE Nijmegen, the Netherlands. Phone: +31-2436-15606. E-mail address: c.verbaarschot@donders.ru.nl (C.S. Verbaarschot).
