# Supplementary material for: Detecting traces of consciousness in the process of intending to act
Source: Exp Brain Res. 2016 Feb 26;234:1945–56. doi: 10.1007/s00221-016-4600-1 (PMC4893062; doi:10.1007/s00221-016-4600-1)
Supplement: Supplementary file 8 — Supplementary material 8 (PDF 1650 kb) [file 221_2016_4600_MOESM8_ESM.pdf]

Experimental Brain Research - Supplementary material -
Detecting traces of consciousness in the process of intending to act

Ceci Verbaarschot [a, 1], Pim Haselager [a] & Jason Farquhar [a]
[a] Donders Institute for Brain, Cognition and Behaviour: Radboud University Nijmegen, the Netherlands.

8 Cluster permutation test on the ERD of the Libet and Matsuhashi task

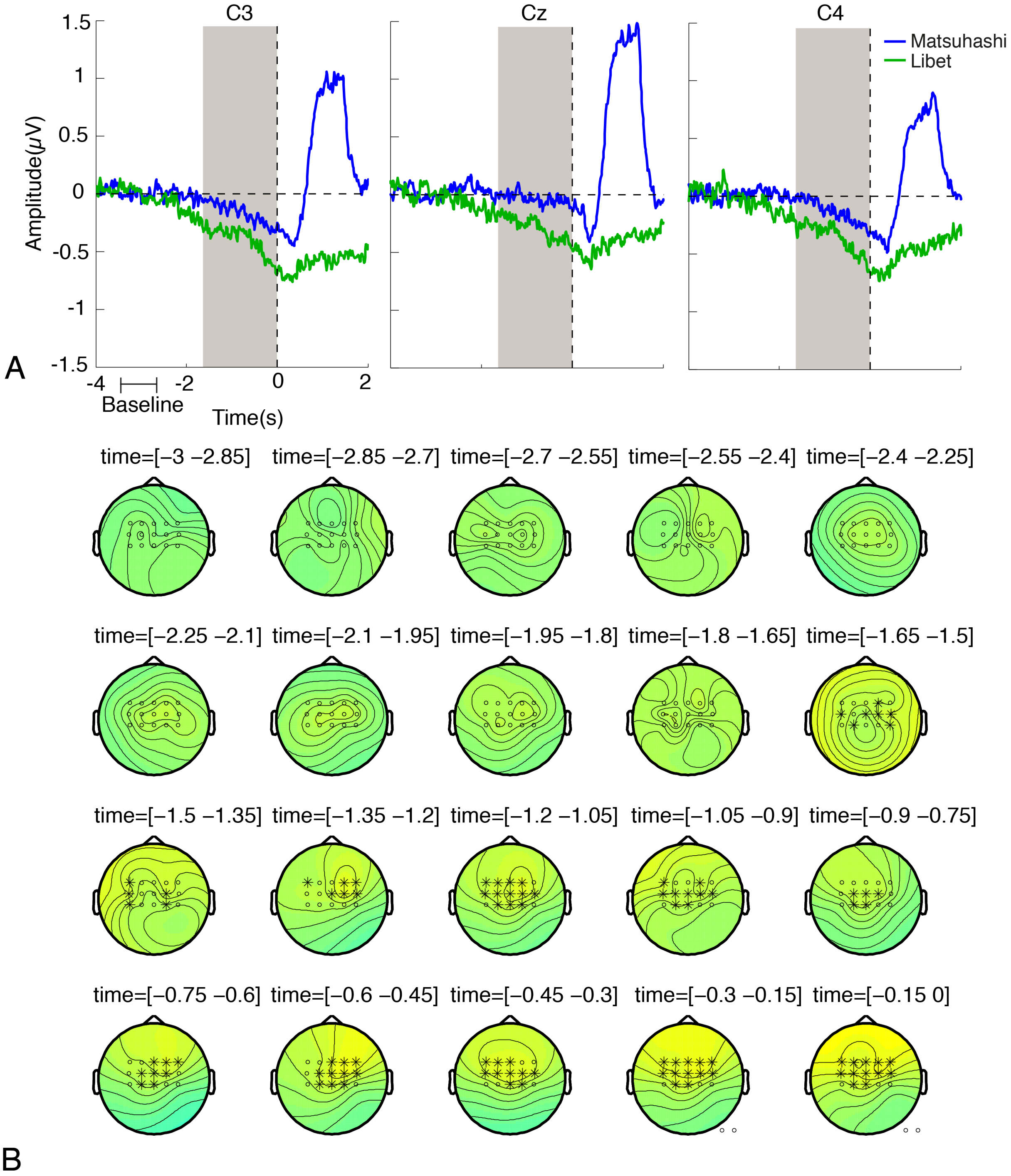

[1] Corresponding author. Address: Center for Cognition, Donders Institute for Brain, Cognition and Behaviour, Radboud University, PO Box 9104, 6500 HE Nijmegen, the Netherlands. Phone: +31-2436-15606. E-mail address: c.verbaarschot@donders.ru.nl (C.S. Verbaarschot).

Fig. 3a *Grand average of the Hilbert transform between 8 and 30 Hz of the Libet and Matsuhashi task. The area shaded in grey indicates the time window (running from -1.65 until 0 s) of the significant cluster identified by the permutation test.* ***b*** *Results of the within-subject cluster permutation test of the Libet and Matsuhashi task. The test was performed on the data of electrodes* Cz, C1, C2, C3, C4, FCz, FC1, FC2, FC3, FC4, CPz, CP1, CP2, CP3 and CP4 *using a time window of -3 to 0 (action onset) s. The electrodes of the identified significant positive cluster are highlighted*
