# Supplementary material for: Detecting traces of consciousness in the process of intending to act
Source: Exp Brain Res. 2016 Feb 26;234:1945–56. doi: 10.1007/s00221-016-4600-1 (PMC4893062; doi:10.1007/s00221-016-4600-1)
Supplement: Supplementary file 9 — Supplementary material 9 (PDF 94 kb) [file 221_2016_4600_MOESM9_ESM.pdf]

Experimental Brain Research

Supplementary material

Detecting traces of consciousness in the process of intending to act

Ceci Verbaarschot [a, 1], Pim Haselager [a] & Jason Farquhar [a]

[a] Donders Institute for Brain, Cognition and Behaviour: Radboud University Nijmegen, the Netherlands.

9 T-tests

Test	Comparison			df	*t*	p
1	Intention Matsuhashi	<	ERD classifier Matsuhashi	8	-2.339	.024*
2	Intention Matsuhashi	<	ERD classifier Libet	11	-2.122	.029*
3	Intention Matsuhashi	<	RP classifier Matsuhashi	11	-2.813	.008*
4	Intention Matsuhashi	≠	RP classifier Libet	11	-1.362	.201
5	Intention Matsuhashi	<	LRP Matsuhashi	10	-3.734	.002*
6	Intention Matsuhashi	<	Intention Libet	11	-8.553	< .001**
7	Intention Matsuhashi	<	Point of no return	11	-8.700	< .001**
8	ERD eye Libet	≠	ERD classifier Libet	11	-0.182	.859
9	ERD eye Libet	<	ERD eye Matsuhashi	8	-5.765	< .001**
10	ERD classifier Matsuhashi	<	ERD eye Matsuhashi	8	-3.901	.002*
11	ERD classifier Matsuhashi	≠	RP classifier Matsuhashi	8	1.082	.311
12	ERD classifier Matsuhashi	≠	LRP Matsuhashi	7	-1.880	.102
13	ERD classifier Matsuhashi	<	Intention Libet	8	-4.598	<.001**
14	ERD classifier Matsuhashi	<	Point of no return	8	-3.970	.002*
15	ERD classifier Libet	≠	ERD classifier Matsuhashi	8	-1.861	.100
16	ERD classifier Libet	≠	RP classifier Libet	11	0.764	.461
17	ERD classifier Libet	<	LRP Libet	7	-2.230	.031*
18	ERD classifier Libet	<	Intention Libet	11	-7.675	<.001**
19	ERD classifier Libet	<	Point of no return	11	-6.064	< .001**
20	RP eye Matsuhashi	≠	RP classifier Matsuhashi	10	-0.748	.111
21	RP eye Matsuhashi	≠	RP eye Libet	8	0.178	.863
22	RP classifier Matsuhashi	≠	RP classifier Libet	11	-0.562	.585
23	RP classifier Matsuhashi	<	LRP Matsuhashi	10	-2.087	.032*
24	RP classifier Matsuhashi	<	Intention Libet	11	-8.273	<.001**
25	RP classifier Matsuhashi	<	Point of no return	11	-8.053	< .001**
26	RP eye Libet	≠	RP classifier Libet	9	-0.470	.650
27	RP classifier Libet	<	LRP Libet	7	-3.302	.007*
28	RP classifier Libet	<	Intention Libet	11	-7.071	<.001**
29	RP classifier Libet	<	Point of no return	11	-6.469	< .001**
30	LRP eye Matsuhashi	≠	LRP eye Libet	6	-1.164	.299
31	LRP Matsuhashi	<	Intention Libet	10	-3.018	.007*
32	LRP Matsuhashi	<	Point of no return	10	-2.565	.014*
33	LRP Libet	<	Intention Libet	7	-1.973	.045*
34	LRP Libet	≠	Point of no return	7	-1.406	.203
35	LRP Libet	<	Intention Libet	7	-1.973	.045*
36	Point of no return	<	Intention Libet	11	-2.104	.030*

Table 2 *Results of the paired-sample (indicated by ≠* or *=) and 1-sided paired sample (indicated by < or >) t-tests. The Bonferroni corrected alpha threshold was set to .001 (i.e. 0.05/36). P-values marked by ** are below the Bonferroni corrected threshold and p-values marked by * are below the normal alpha threshold of 0.05*

[1] Corresponding author. Address: Center for Cognition, Donders Institute for Brain, Cognition and Behaviour, Radboud University, PO Box 9104, 6500 HE Nijmegen, the Netherlands. Phone: +31-2436-15606. E-mail address: c.verbaarschot@donders.ru.nl (C.S. Verbaarschot).
