# Supplementary material for: Detecting traces of consciousness in the process of intending to act
Source: Exp Brain Res. 2016 Feb 26;234:1945–56. doi: 10.1007/s00221-016-4600-1 (PMC4893062; doi:10.1007/s00221-016-4600-1)
Supplement: Supplementary file 10 — Supplementary material 10 (PDF 202 kb) [file 221_2016_4600_MOESM10_ESM.pdf]

Experimental Brain Research

Supplementary material
Detecting traces of consciousness in the process of intending to act

Ceci Verbaarschot [a, 1], Pim Haselager [a] & Jason Farquhar [a]

[a] Donders Institute for Brain, Cognition and Behaviour: Radboud University Nijmegen, the Netherlands.

10 Box-and-whisker plot

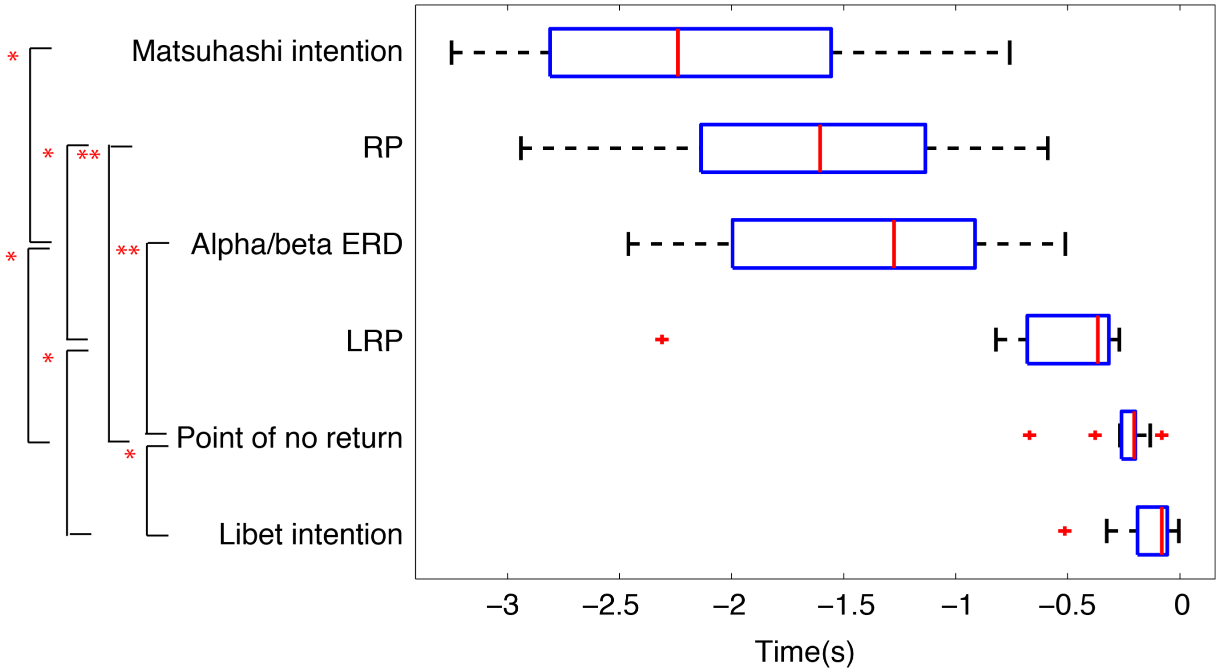

Fig. 4 A box-and-whisker plot of the estimated RP, LRP, alpha/beta ERD and intention onsets over all participants. The boxes denote the first and third quartiles of the data and the whiskers extend to the most extreme data points (outliers are denoted by a red cross). The median is denoted by a vertical line inside the box. The RP and alpha/beta ERD boxes incorporate the onsets estimated by classifier for the Libet task. Similarly, the LRP box incorporates the estimated LRP onsets by eye for the Libet task. A red star indicates that the estimated onsets differed significantly between the indicated groups with $p < .05$. Two red stars indicate a significance of $p < .001$.

[1] Corresponding author. Address: Center for Cognition, Donders Institute for Brain, Cognition and Behaviour, Radboud University, PO Box 9104, 6500 HE Nijmegen, the Netherlands. Phone: +31-2436-15606. E-mail address: c.verbaarschot@donders.ru.nl (C.S. Verbaarschot).
